# Effect of acupuncture and its influence on cerebral activity in functional dyspepsia patients: study protocol for a randomized controlled trial

**DOI:** 10.1186/s13063-016-1296-2

**Published:** 2016-04-02

**Authors:** Seok-Jae Ko, Kyungmo Park, Jieun Kim, Minji Kim, Joo-Hee Kim, Jeungchan Lee, Abdalla Z. Mohamed, Inkwon Yeo, Jinsung Kim, Sun-Mi Choi, Honggeol Kim, Jae-Woo Park, Jun-Hwan Lee

**Affiliations:** Department of Gastroenterology, College of Korean Medicine, Kyung Hee University, Seoul, Republic of Korea; Department of Biomedical Engineering, Kyung Hee University, Yongin, Republic of Korea; Division of Clinical Research, Korea Institute of Oriental Medicine, Daejeon, Republic of Korea; Department of Statistics, Sookmyung Women’s University, Seoul, Republic of Korea; Department of Clinical Korean Medicine, Graduate School, Kyung Hee University, Seoul, Republic of Korea; Athinoula A Martinos Center for Biomedical Imaging, Department of Radiology, Massachusetts General Hospital, Harvard Medical School, Charlestown, USA; Korean Medicine Life Science, University of Science & Technology, Campus of Korea Institute of Oriental Medicine, Daejeon, Republic of Korea

**Keywords:** Functional dyspepsia, Acupuncture, Streitberger needle, Functional magnetic resonance imaging, Metabolomics

## Abstract

**Background:**

Functional dyspepsia (FD) is a prevalent gastric disorder that is difficult to manage due to lack of satisfactory treatments. Acupuncture has been studied with regard to the rising need for treating FD, but the mechanism verifying its efficacy has not yet been fully revealed. The aim of this study is to explore the efficacy and mechanism of acupuncture for FD compared with a sham group.

**Methods/design:**

We describe a proposal for a randomized, assessor-blind, sham-controlled trial with 70 eligible participants who will be randomly allocated either into an acupuncture or a sham group. Participants in the acupuncture group will receive 10 sessions of real acupuncture treatment and those in the sham group will be treated with identical sessions using a Streitberger needle. Functional magnetic resonance imaging (fMRI) and metabolomics studies will be implemented before and after 4 weeks of treatment to investigate the mechanism of acupuncture. The primary outcome is a proportion of responders with adequate symptom relief and the secondary outcomes include the Nepean Dyspepsia Index - Korean version, Functional Dyspepsia-Related Quality of Life questionnaire, Ways of Coping Questionnaire, Coping Strategies Questionnaire, perception of bodily sensation questionnaire, State-Trait Anxiety Inventory, and the Center for Epidemiological Studies - Depression Scale. The outcomes will be evaluated before and after the treatment.

**Discussion:**

This is the first large-scale trial evaluating the efficacy and mechanism of acupuncture with fMRI and metabolomic methods. We will compare real acupuncture with the Streitberger sham needle to verify the specific effect of acupuncture. The results of this trial are expected to be relevant evidences affecting policy and decision-makers associated with routine healthcare.

**Trial registration:**

ClinicalTrials.gov Identifier: NCT02358486.

Date of Registration: 21 January 2015.

## Background

Functional dyspepsia (FD) is a common gastrointestinal disorder that is defined as persistent or recurrent abdominal pain or discomfort centered in the upper abdomen that occurs in the absence of any structural lesion [1]. The prevalence of FD varies from 11 % to 29.2 % globally [2], and recently more than 40 % of patients who visited hospitals in South Korea have been diagnosed with FD [3]. The conventional treatments for FD include antisecretory agents, prokinetics, and antidepressant agents [4]. However, owing to the unsatisfactory efficacy of these approaches, patients with FD often turn to alternative and complementary therapies such as acupuncture [5]. Acupuncture has been used in Asia for centuries to treat FD. During the past decade, many clinical and experimental research studies have revealed that acupuncture reduces gastric symptoms, such as abdominal distension, epigastric pain, and belching [6–8]. A recent meta-analysis on acupuncture for FD reported that there was a lack of robust evidence on the efficacy and safety of acupuncture, which might be due to the poor quality of previous studies [9]. The mechanism and efficacy of acupuncture is still not fully understood, and high-quality studies with a suitable design for acupuncture are needed.

Positron emission tomography (PET) and functional magnetic resonance imaging (fMRI) are promising techniques for exploring the central mechanism of acupuncture [10]. A recent study indicated that the regional and distant connectivity of the anterior cingulate cortex and thalamus are correlated with the severity of FD [11]. Authentic and sham acupuncture procedures have relatively different brain responses in the insula and hypothalamus [12]; therefore, an emphasis has recently been placed on differentiating the brain areas activated during acupuncture.

Metabolomics is defined as the systematic study to measure the multi-parametric metabolic response of a living system to pathophysiological stimuli or genetic modification quantitatively [13, 14]. In contrast to the reductionism of modern medicine, a metabolomic study approaches from the viewpoint of a living organism in systemic way, which corresponds with the traditional Korean medicine (TKM) theory that focuses on holistic treatment. In addition, changes in metabolites reflect the response of the biological system; therefore, metabolomics could provide insights about possible mechanisms underlying the effects of acupuncture. A recent nuclear magnetic resonance (NMR)-based metabolomics study showed the potential biological effect of acupuncture on FD [15]; however, there are some limitations, and the scientific evidence is still insufficient.

Considering these features, a randomized, assessor-blind, sham-controlled clinical trial is planned to investigate the effect of acupuncture on FD. To analyze the mechanism of acupuncture, fMRI will be used in the study to identify the differences between the sham and acupuncture groups and their influence on clinical outcomes. We will also use 1H NMR-based metabolomics to characterize the difference in the metabolic profiles of the plasma and the urine of FD patients in the acupuncture group compared with the sham control group.

## Methods/design

### Objectives

The aims of this study are as follows:

1. Investigate whether manual acupuncture brings adequate relief of FD symptoms in comparison with the sham control group.
2. Determine the difference of brain activity between the real and sham acupuncture groups and the correlation between brain and clinical outcomes.
3. Assess any correlation between differences and changes in the metabolite and clinical outcomes.

### Hypothesis

We hypothesized that 10 sessions (4 weeks) of acupuncture treatment will improve FD symptoms, and a mechanistic difference will exist between the acupuncture and the sham control for FD based upon the fMRI and the metabolomics study.

### Design

**Fig. 1:**
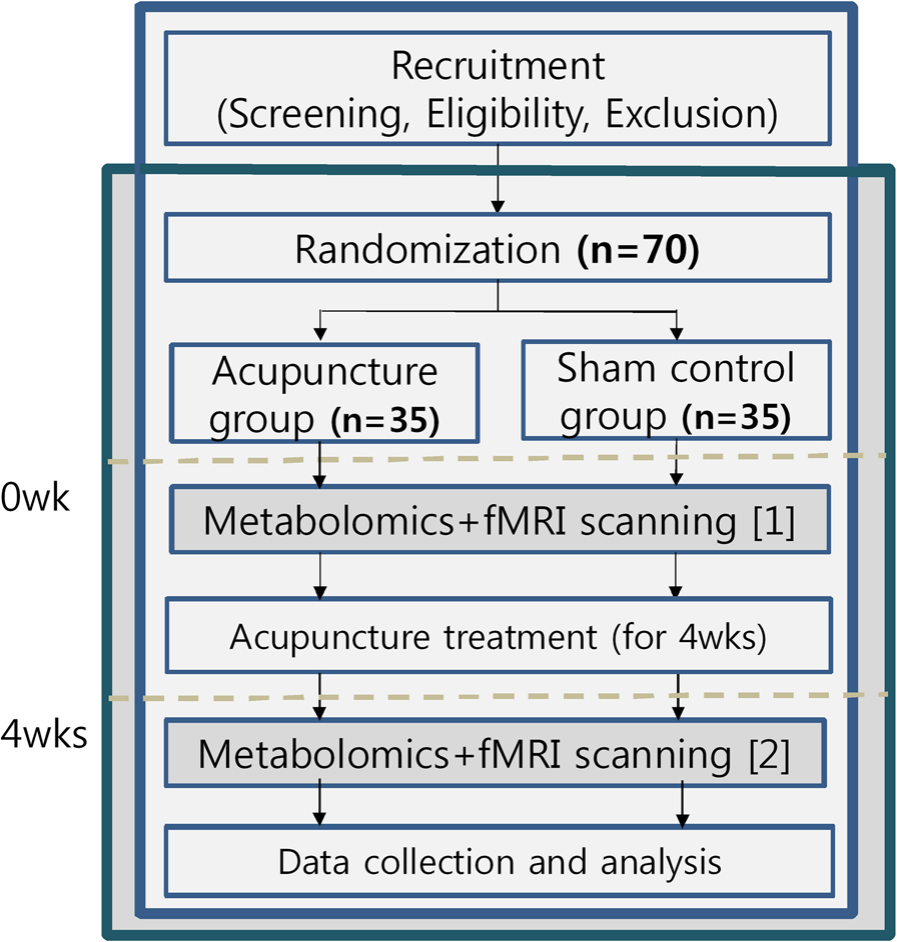
Flow of the study

A randomized, assessor-blind, sham-controlled trial will be performed at the Kyung Hee University Hospital at Gangdong in Seoul, Korea, beginning in January 2015. The flow of the entire trial is shown in Fig. 1.

### Ethical considerations

This trial will be carried out along with the protocol approved by the Institutional Review Board (IRB) of the Kyung Hee University Hospital at Gangdong (KHNMCOH 2014-08-002-002), the standards of the International Committee on Harmonization on Good Clinical Practice, and the revised version of the Declaration of Helsinki. The purpose and risks of the trial will be fully explained to the participants, who will be required to provide informed consent to indicate that they agree with the protocol and would take part in the trial. The participants will be able to quit at any time during the study period. Any financial costs incurred due to adverse events related to the trial will be covered for the participants. This trial is registered at ClinicalTrials.gov (NCT02358486).

### Inclusion and exclusion criteria

The inclusion and exclusion criteria are based on a previous study [16].

#### Inclusion criteria

The inclusion criteria are as follows:

1. Age between 30 and 49 years.
2. Meeting the definition of FD and postprandial distress syndrome (PDS) as a subtype of FD according to the Rome III criteria [17].
3. Complaints of a degree of dyspepsia that produces a score of more than 40 points on the visual analog scale (VAS; 0, no symptom at all; 100, the most severe symptom one has ever had).
4. Answer at least three questions worth more than 2 points on the Gastrointestinal Impact Scale.
5. Have normal esophagogastroduodenoscopy results within a year and no evidence of any organic diseases.
6. Individuals who are not supposed to take any other treatments associated with FD during the study.

#### Exclusion criteria

Participants with the following characteristics will be excluded:

1. Individuals who have obvious organic diseases such as reflux esophagitis.
2. Individuals who have apparent signs of irritable bowel syndrome.
3. Individuals who have alarming symptoms (weight loss, black or tarry stool, or dysphagia).
4. Individuals who have serious internal organ diseases (diseases of the heart, lung, liver, or kidney) or mental illness.
5. Individuals who have undergone surgery related to the gastrointestinal tract.
6. Individuals who are pregnant or breastfeeding.
7. Individuals who are taking drugs that could affect the gastrointestinal tract; a minimum wash-out period of 2 weeks is required before participating in the trial.
8. Individuals who are positive for human immunodeficiency virus infection.
9. Individuals who have severe problems due to malabsorption.
10. Individuals who have difficulties in attending the trial (for example, due to paralysis, serious mental illness, dementia, drug addiction, time constraints, severe disorder of vision or hearing, or illiteracy).
11. Individuals with other issues that could interfere with acupuncture treatment (for example, clotting disorders or leukopenia, a pacemaker, epilepsy, or anticoagulant therapy).
12. Individuals who have metal implants or fragments that might influence the fMRI examination.

### Recruitment

Banner advertisements will be placed on the notice boards in the hospital. We will run classified advertisements on web sites that patients with digestive problems often visit. We will place the advertisements in the local newspaper and at the bus and subway stations.

### Randomization, allocation concealment, and blinding

The randomization will be carried out using random number lists created in accordance with the PROC PLAN of SAS 9.2 (SAS Institute Inc., Cary, NC, USA) by an independent statistician (IY). The allocation list will be handled by an independent researcher, who will provide random numbers to the investigator at the hospital via email. A random number will be assigned sequentially when the participants pass the screening test. Investigators who have contact with the participants should be unaware of the random allocation. Participants and assessors will be blinded as to which group they are assigned; however, practitioners involved in the study will not be blinded. The allocation and feedback emails will be saved, and the unblinding procedure will be documented and stored in the trial master file. The success of the blinding will be evaluated after the completion of the trial.

### Intervention

Detailed information regarding the acupuncture treatment is described in the form of the revised STandards for Reporting Interventions in Clinical Trials of Acupuncture (STRICTA) [18] listed in Table 1. Thirteen acupoints that are widely used to manage digestive symptoms are selected [19, 20]. The treatment will last for 20 minutes each visit, and a total of ten treatment session will be implemented, including two sessions during the MRI scanning.

**Table 1:** Acupuncture treatment details based on the STRICTA 2010 checklist

Item	Detail
1. Acupuncture rationale	1a) Style of acupuncture
	- Manual acupuncture based on traditional meridian theory.
	1b) Reasoning for treatment provided, based on historical context, literature sources, and/or consensus methods, with references where appropriate
	- Manual acupuncture treatments based on the traditional meridian theory, clinical experience, and consensus by the experts in acupuncture and FD.
	1c) Extent to which treatment was varied
	- No additional acupoints allowed.
2. Details of needling	2a) Number of needle insertions per subject per session (mean and range where relevant)
	- Fixed 13 acupoints.
	2b) Names (or location if no standard name) of points used (uni/bilateral)
	- LI 4, ST36, LR3, PC3, SP4, and ST34 (bilateral) and CV12 (unilateral).
	2c) Depth of insertion, based on a specified unit of measurement or on a particular tissue level
	- From 5 to 30 mm.
	2d) Response sought (for example, de qi or muscle twitch response)
	- De qi sensation
	2e) Needle stimulation (manual, electrical)
	- Manual stimulation: needle rotation with thumb and index fingers at 3 Hz.
	2f) Needle retention time
	- Fifteen minutes.
	2 g) Needle type (diameter, length, and manufacturer or material)
	- A sterilized stainless steel needle (0.2 × 40 mm, Dongbang Acupuncture Inc., Ungcheon, Boryeong, Korea).
3. Treatment regimen	3a) Number of treatment sessions
	- Ten treatment sessions in both groups including two sessions during fMRI scanning.
	3b) Frequency and duration of treatment sessions
	- Twice weekly for 4 weeks plus additional two treatments during fMRI scanning (before and after treatment), 15 minutes for each session.
4. Other components of treatment	4a) Details of other interventions administered to the acupuncture group (moxibustion, cupping, herbs, exercises, lifestyle advice)
	- No other interventions during the study period allowed.
	4b) Setting and context of treatment, including instructions to practitioners, and information and explanations to patients
	- Participants will be informed that the acupuncture treatment is based on traditional Korean medicine and previous studies on clinical trial.
5. Practitioner background	5) Description of participating acupuncturists (qualification or professional affiliation, years in acupuncture practice, other relevant experience)
	- Korean medicine doctors who have license and at least 3 years of experience of treating gastrointestinal diseases. They have studied acupuncture for more than 10 years and graduated from a university of Korean medicine. To ensure providing identical treatments, they finished 10 hours of training and simulated the protocol.
6. Control or comparator interventions	6a) Rationale for the control or comparator in the context of the research question, with sources that justify this choice
	- Streitberger needle will be used as a sham control (its rationale is described in background and discussion of main text).
	6b) Precise description of the control or comparator. If sham acupuncture or any other type of acupuncture-like control is used, provide details as for items 1 through 3 above.
	- Practitioners will use identical acupoints to sham group compared with real acupuncture group.
	- The depth of insertion, response sought, needle stimulation, needle retention time, treatment period, and number of treatment sessions in sham group will be the same as those in real acupuncture group.
	- In sham acupuncture group, Streitberger placebo needles (0.2 × 40 mm, Asia Med GmbH, Germany) will be used.

*STRICTA* STandards for Reporting Interventions in Clinical Trials of Acupuncture, *FD* functional dyspepsia, *LI* large intestine, *ST* stomach, *LR* liver, *PC* pericardium, *SP* spleen, *CV* conception vessel

### fMRI scanning procedure

fMRI data will be acquired using a 3 T Philips Achieva MRI scanner (Philips Medical System, Best, the Netherlands) equipped for echo planar imaging with an 8-channel head coil. A whole-brain T2*-weighted gradient echo blood oxygenation level-dependent (BOLD) pulse sequence (repetition time [TR]/echo time [TE] = 2000/35 ms, flip angle = 90°, 34 axial slices, voxel size = 2.87 × 2.87 × 4 mm^3^) will be used. In addition to the fMRI data, we will collect structural data using a T1-weighted 3D turbo field echo (TR/TE = 9.9/4.6 ms, flip angle = 8°, voxel size = 1 mm isotropic). Patients will be asked to lay supine in the scanner while wearing earplugs to attenuate the gradient noise.

Patients will participate in three sessions on the same day: a training session, a nutrition drink test session, and an imaging session. The training session is intended to familiarize the subjects with the modified pressure algometer (MA) and the rating procedures. For the nutrition drink test session, we will induce postprandial symptoms and determine pressures to produce appropriate abdominal discomfort to be used during the subsequent imaging session.

1. In the training session, we will introduce our pressure-induced discomfort stimuli and rating procedures. A series of 14-second pressure stimuli will be delivered to the epigastric area using an MA connected to a rapid cuff inflator system (Hokanson E20, AG101, Bellevue, WA, USA). This rapid cuff inflation system allows us to set an accurate pressure within 1 mmHg. Using this system, the target pressure can be reached in 2 seconds, and we can deflate the pressure immediately. Subjects will be asked to verbally rate their pressure-induced discomfort using a 0–100 numerical scale, where 0 is labeled “no discomfort” and 100 is labeled “the worst discomfort, but not painful.” We will set the abdominal pressure to target a discomfort intensity of about 60/100 as “moderate discomfort.” The pressure required to induce a 60/100 discomfort intensity (D60) and discomfort symptoms from the abdominal pressure will be recorded during the training session.
2. In the nutrition drinking test session, subjects will be asked to consume canned drinks in the morning after an overnight fast. We will use the same procedures as those described in a previously reported protocol [21]. Subjects will consume 200 mL of Newcare (Welife, Cheonan, Republic of Korea), which contains 1 kcal/mL with 20 g of carbohydrates, 6 g of fat, and 7 g of protein. Subjects will drink a can every 4 minutes until they reach a predetermined maximum level of fullness. At 4-minute intervals, the subjects will verbally rate their overall fullness levels on a range of 0–5 (0: no fullness, 1: the first sensation of fullness, 2: mild, 3: moderate, 4: severe, and 5: unbearable fullness). The participants will be instructed to stop drinking when their fullness reaches a level of 4. The actual volume of the canned drinks consumed up until this point is the maximum tolerable volume. Questionnaires for postprandial symptoms, intensity of abdominal discomfort, and abdominal pressure for the D60 will be administered 15 minutes after participants complete the test. The regained pressure for D60 at this point will be used during the imaging session.
3. In the imaging session, fMRI data will be acquired for the meal-loaded discomfort resting state run (REST), continuous pressure stimuli run (CONT), and block design pressure stimuli run both before acupuncture (TASK1) and after acupuncture (TASK2). Following each scan, the subjects will be asked to verbally rate their discomfort intensity and fullness level after each run using the same scales (a 0–100 scale for the discomfort intensity, a 0–5 scale for the fullness level) as previously instructed during the training and nutrition drinks test sessions.

For runs in both the REST and CONT runs, subjects will be instructed to relax and lie still with their eyes open. The REST is a 6-minute run, and subjects with abdominal discomfort induced by the nutrition drink test, as described above, will be allowed to rest without pressure stimuli. The CONT (continuous MA-induced discomfort state) lasts for 7 minutes, and a constant and individually tailored pressure (D60) will be delivered to each subject.

**Fig. 2:**
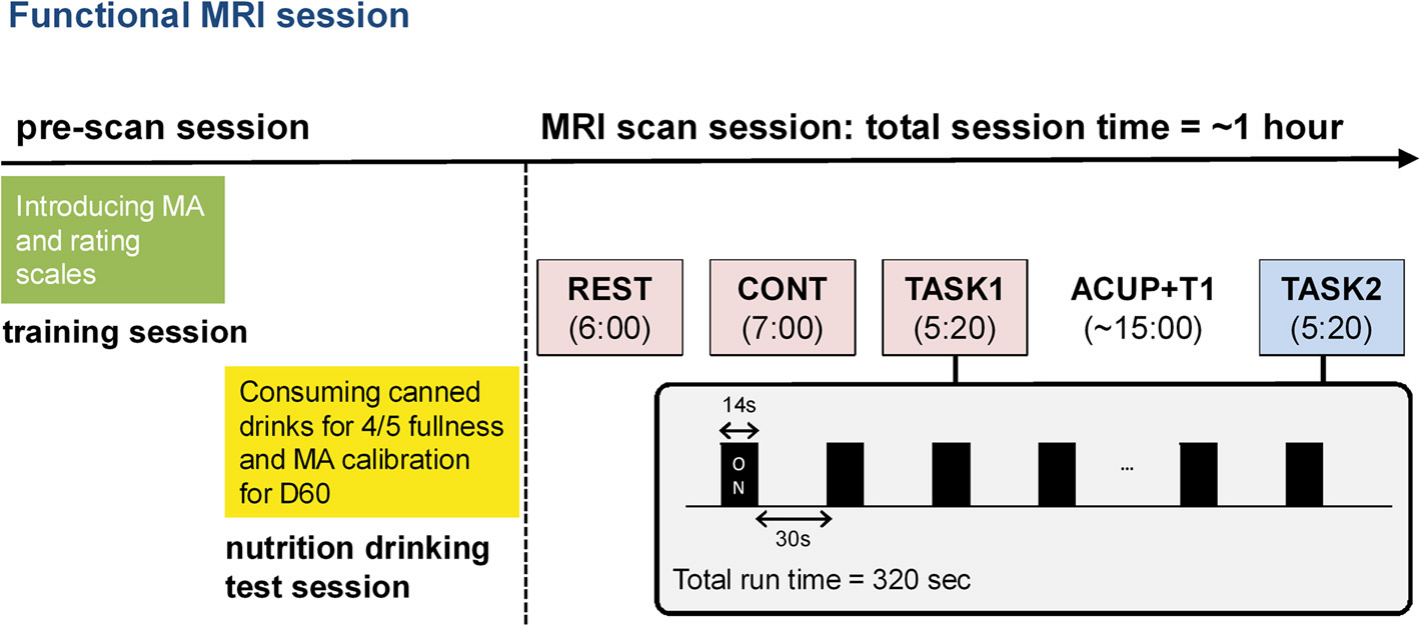
Functional magnetic resonance imaging experiment paradigm (REST, meal-loaded steady-state resting run; CONT, continuous MA steady-state run; TASKT1/2, block design MA stimulus run; T1, structural image acquisition; ACUP, acupuncture treatment; MA, modified pressure algometer)

Both TASK1 and TASK2 runs will consist of a block design with seven 14-second stimulation blocks (ON) interspersed between eight 30-second rest blocks (OFF). The total time for the block design run is 5 minutes and 20 seconds. For the stimulation blocks, we will use the same D60 pressure level, which is predetermined from the nutrition drinks test session. The subjects will be instructed to use a button during the TASK runs to indicate when the pressure-induced discomfort is not decreasing after the abdominal pressure stimulation ends. Either real or sham acupuncture will be applied after the TASK1 run, and 15 minutes of retention time will be given to gain a sufficient acupuncture effect. The TASK2 run will be followed by the acupuncture treatment and structural data collection. The entire imaging session procedure is summarized in Fig. 2.

### fMRI data processing

Functional MRI data will be processed using the FSL (FMRIB Software Library; http://fsl.fmrib.ox.ac.uk/fsl), AFNI (Analysis of Functional NeuroImages; https://afni.nimh.nih.gov/afni), and FreeSurfer (http://freesurfer.net) software packages. Data will be corrected for physiological artifacts, slice timing, and affine head motion, and non-brain extraction will also be performed. Cortical surface reconstruction will be carried out to improve structural-functional co-registration using FreeSurfer’s bbregister tool, and then the data will be registered to the standard Montreal Neurological Institute (MNI) space. Spatial smoothing and temporal filtering will be applied.

We will determine the functional connectivity for the data from the REST and CONT runs using seed correlation analysis and independent component analysis. For the seed correlation analysis, seeds will be defined based on the block design runs results (TASK1 and TASK2) and other discomfort/visceral stimulation fMRI studies [22–25]. For the TASK runs, statistical parametric mapping at the single subject level will be carried out using a generalized linear model (GLM) with a stimulation block design and discomfort as the explanatory variables. In order to investigate the link between discomfort-induced brain functions and behavioral/clinical measures, we will perform a whole-brain voxel-wise linear analysis. All brain maps will be thresholded using a cluster correction for multiple comparisons at a z score > 2.3 and a cluster size threshold of *p* < 0.05.

### Outcome measurement

A clinical research coordinator who is also a licensed nurse will evaluate the outcome variables as an assessor. He/she is supposed to be unaware of the group to which the subject is assigned.

#### Primary outcome

The primary outcome is the proportion of responders (PR) [26] defined as the proportion of participants who answer “yes” to more than half of the adequate relief questions during the treatment period. The adequate relief question is: After the last visit, have you had adequate relief of your stomach pain or discomfort?

#### Secondary outcomes

##### Nepean Dyspepsia Index - Korean version (NDI-K)

The Nepean Dyspepsia Index (NDI) was developed by Talley et al. [27] and has been reported as a reliable questionnaire to measure the severity of dyspeptic symptoms and quality of life [28, 29]. The Korean version of the NDI (NDI-K) was validated by Lee et al. [30]. The minimum clinically important difference of NDI is 10 points on the total scale [31]. The NDI-K consists of two categories (symptom-based questions and quality of life); in this study, we will use only symptom-based questions about the period, severity, and degree of distress of 15 symptoms at baseline and again after 4 weeks.

##### Functional Dyspepsia-Related Quality of Life (FD-QoL) questionnaire

The FD-QoL questionnaire is made up of four sections with a total of 21 questions regarding quality of life measured by a 5-point Likert scale (0: not at all or not applicable, 1: a little, 2: moderately, 3: quite a lot, and 4: extremely). The FD-QoL questionnaire is distributed into 5 items about diet, 4 items about daily activity, 6 items regarding emotion, and 6 items about social functioning. The questionnaire is known to be reliable and trustworthy for assessing the effect of dyspepsia on a patient’s overall quality of life [32]. The FD-QoL questionnaire will be administered at baseline and again after 4 weeks.

##### Numeric Rating Scale (NRS) for abdominal discomfort

Subjects will be asked to verbally rate the intensity of their abdominal discomfort after each MA-induced discomfort MRI run (CONT, TASK1, and TASK2). We will use a 0–100 Numeric Rating Scale (NRS), where 0 is labeled “no discomfort” and 100 is labeled “the most intense discomfort tolerable”.

##### Ways of Coping Questionnaire (WCQ)

We will use the WCQ, which is composed of 66 items and uses a 4-point Likert scale that was developed by Folkman et al. [33] and validated by Lundqvist et al. [34]. There are 8 coping strategies: (1) Confrontive coping, (2) Distancing, (3) Self-controlling, (4) Seeking social support, (5) Accepting responsibility, (6) Escape-avoidance, (7) Planful problem solving, and (8) Positive reappraisal. In the WCQ, these 8 areas are categorized into 2 groups (active coping and passive coping). The WCQ will be administered at baseline and again after 4 weeks.

##### Coping Strategies Questionnaire (CSQ)

The CSQ is made up of 48 items and uses a 7-point Likert scale validated by Franco et al. [35] to evaluate the coping strategy for pain or acupuncture treatment. The CSQ is distributed across 8 areas: (1) Catastrophizing, (2) Distractor behaviors, (3) Self-instructions, (4) Ignoring the pain, (5) Reinterpreting the pain, (6) Hoping, (7) Faith and praying, and (8) Cognitive distraction. These areas are classified as either adaptive coping or disadaptive coping. The CSQ will be administered at baseline and after 4 weeks.

##### Perception of bodily sensation (PBS)

We will apply the PBS questionnaire developed by Schneider et al. [36] at baseline to assess the response to the abdominal discomfort induced by MA. The level of perceived bodily sensation varies from person to person and might lead to different responses of brain activation. The questionnaire consists of 10 areas and uses a 6-point Likert scale where higher points indicate a more sensitive response. In our trial, the PBS plays an important role in analyzing the brain’s response to abdominal discomfort.

##### State-Trait Anxiety Inventory (STAI)

The STAI [37] is a questionnaire that evaluates anxiety as a psychological factor. It consists of 40 items: 20 items to assess the state of anxiety (anxiety triggered by a specific event), and 20 items to determine the trait of anxiety (anxiety derived from personal characteristics) according to a 4-point Likert scale. Higher scores indicate a higher degree of anxiety. Dyspeptic symptoms and anxiety are closely correlated [38, 39]. Therefore, the effect of acupuncture on anxiety will be evaluated. This measurement will be checked at baseline and again after 4 weeks.

##### Center for Epidemiological Studies - Depression Scale (CES-D)

The CES-D was developed by Radloff in 1977 and has been applied worldwide [40, 41]. It is both valid and reliable for assessing self-reported depression. The CES-D is composed of 20 areas; it uses a 4-point Likert scale where higher scores indicate more severe depression. Generally, a total sum score of more than 25 points indicates definite depression; more than 21 points is probable depression, while more than 16 points is possible depression. The CES-D will be evaluated at baseline and after 4 weeks to determine if there was an association between depression and the effects of acupuncture treatment.

##### Metabolomics

A metabolomics study will be performed to analyze the difference between (1) FD before and after acupuncture treatment and (2) the acupuncture and sham acupuncture groups. Blood and urine samples will be collected at baseline and after 4 weeks. After collecting the blood, the plasma will be isolated by centrifugation and stored in a −73 °C deep freezer. The urine samples will be inverted two or three times, pipetted into the cryo tubes, and stored in the deep freezer until the time of analysis. The sample will be analyzed by NMR and ultra-performance liquid chromatography quadrupole/time of flight mass spectrometry (UPLC-Q/TOF MS). After obtaining the NMR and MS data for blood and urine samples, the patterns and biomarkers for FD could be discovered using a multivariate statistical analysis.

### Sample size calculation

The sample size was calculated based on a previous study [42] and the opinions of an expert group. Considering the previous study and the fact that more than a 30 % difference is regarded as significant in clinical practice based upon expert opinions, we assume that 70 % of the participants in the acupuncture arm would achieve adequate relief for over half of the study period, while up to 35 % of those in the sham group would achieve such an outcome. We set the dropout ratio at 10 %, the level of significance at α = 0.05, and a power of 1-ß = 0.80. The sample size was estimated based on the following formula:

$$ \mathrm{n}={\left({\mathrm{z}}_{\alpha /2}\sqrt{2\overline{p}\left(1-\overline{p}\right)}+\mathrm{z}\upbeta \sqrt{p_t\left(1-{p}_t\right)+{p}_c\left(1-{p}_c\right)}\right)}^2/{\left({p}_t-{p}_c\right)}^2 $$

assuming p_t_ = 0.7 (p_t_: the effect of the acupuncture group) and p_c_ = 0.35 (p_c_: the effect of the sham control group).

We also considered the recent trend of neuroimaging trials when calculating sample size. In fMRI studies that use approximately 90,000 voxels to estimate the BOLD signal indirectly, conventional power calculations are often meaningless. Therefore, most related studies determined less than 20 participants to be an adequate sample size [22, 43, 44] due to the high cost of fMRI examinations. Therefore, we have assumed 35 subjects in each group considering the global trend of increases in subjects to ensure that we obtain sufficient data for our analysis.

### Statistical analysis

A statistical analysis will be conducted by an independent statistician using SPSS 16.0 (SPSS Inc., Chicago, IL, USA). All continuous variables will be presented as means and SDs, and categorical variables will be shown as a number (%). All analyses will be based on the intention-to-treat principle using the last observation carried forward rule. The statistical significance level will be considered to be 0.05 (two-sided) with 95 % confidence intervals (CI). When continuous variables are compared between two groups, a two-sample *t*-test (parametric method) or Mann–Whitney *U* test (non-parametric method) will be used. The paired *t*-test (parametric method) or Wilcoxon signed ranks test (non-parametric method) will be used to compare continuous variables both before and after the treatment. When the variable is categorical, a chi-square test or Fisher’s exact test will be implemented. If there are statistically significant differences between groups, an analysis of covariance (ANCOVA) or logistic regression will be used.

#### Metabolomics study analysis

A multivariate statistical analysis will be conducted from the data obtained from the MS or NMR spectra of collected blood or urine samples using SIMCA-P version 12.0 (Umetrics, Umeå, Sweden). A principal component analysis (PCA), partial least squares-discriminant analysis (PLS-DA), or orthogonal projections to latent structure-discriminant analysis (OPLS-DA) will be used to process the data. In addition, to investigate the variables affecting the differences in metabolites between the groups, the loading plot, S-plot, or the variable influence on projection (VIP) value will be used.

### Safety

We will conduct the following tests on all participants at the screening stage to exclude patients with serious organic lesions: white blood cell, hemoglobin, hematocrit, platelet, aspartate aminotransferase/alanine aminotransferase, gamma-glutamyl transpeptidase, blood urea nitrogen, creatinine, and erythrocyte sedimentation rate. Adverse events (AEs) will be thoroughly documented at each visit, and the time of occurrence, severity, treatment, and progress will be recorded. The principal investigator will evaluate any associations between AEs and the trial. If serious AEs occur, they will be reported to the IRB immediately. Participants will be given notice of any cautions and possible side effects before the fMRI examination. If side effects develop because of the acupuncture treatment, such as skin lesions, subcutaneous bruising, bleeding or phlebitis signs, participants will be treated appropriately at no cost to them.

### Quality control

To maintain a consistent high quality of the clinical trial, the clinical research associate will monitor the study file, informed consent forms, case report forms, serious AEs, and data records regularly.

## Discussion

This study is planned as a randomized, assessor-blind, sham-controlled trial on the efficacy of acupuncture treatment of PDS in FD diagnosed using the Rome III criteria compared with the sham acupuncture group. Our trial is one of the largest randomized controlled trials to explore the mechanism of acupuncture for FD using fMRI examination and metabolomics study. This study is also the first study to investigate the influence of acupuncture on abdominal discomfort induced by a meal and the MA pressure-loading device during MRI scanning.

The researchers who have conducted previous acupuncture studies have invested a great amount of effort into developing appropriate control groups. Creating proper control groups in acupuncture trials is crucial, because blinding for practitioners is virtually impossible. In order to design an appropriate control, we will have to consider the three major and commonly accepted therapeutic effects of acupuncture treatment: point-specific effect, placebo, and non-specific physiological effect [45]. Acupuncture trials have focused on finding evidence that the acupuncture treatment is more than the accumulated non-specific effects related to simple needle insertion. However, due to the fact that acupuncture is a physical, invasive, and manual procedure, separating both the specific and non-specific effects of acupuncture is extremely challenging. To differentiate specific from non-specific effects, researchers have focused on non-acupoints as a sham control [9], but none of the trials have mimicked manual acupuncture in a real clinical practice or adequately fulfilled the criteria of being truly inert [46]. Our previous study [16], which had a similar design as this trial, used the acupuncture wait-list group as the control group. As a result, the acupuncture group showed a significantly superior effect over the wait-list participants after expectation and patient-doctor augmentation effects were controlled for to exclude a placebo effect (data not shown). However, there were limitations, such as the non-specific effects that simply resulted from the needle’s insertion. To overcome these issues, we will use Streitberger needles for the patients in the sham control group. In their study, Streitberger et al. reported that none of the volunteers suspected that the needle might not have penetrated the skin [47]. This type of needle has been regarded as a reliable sham control in many studies [48–50] and produced successful blinding even in Chinese subjects who had prior experience with was estimated based on the acupuncture [51]. We will use various methods to increase credibility, such as keeping the sham needles out of the patients’ direct view and using 12-mm wide Steri-strip (manufactured by 3 M) and MRI-compatible needles to fix the needle evenly inside the foot, including SP4, in the MRI scanner. The success of these attempts at blinding will be examined after the trial’s conclusion.

Current neuroscience studies using positron emission tomography-computed tomography on FD have demonstrated an association with the cerebral cortex. The anterior cingulate cortex (ACC), insula, and thalamus/hypothalamus, which are the key regions of “gut-brain communication,” might be most closely related to the severity of FD [52]. A recent report revealed that acupuncture might affect the activity of somatic and visceral sensation and therefore modulate the insula, ACC, and hypothalamus, which are related to the regulation of homeostasis [10]. One previous neuroimaging study reported a central mechanism of acupuncture associated with the pain and anxiety regions of the brain after gastric distension was induced by inserting a rubber balloon into the subjects’ stomach [53]. However, the methods of inducing gastric distension were considered to be inappropriate because they were inconvenient and invasive, and there was no intervention, such as acupuncture. Our study is the first neuroimaging study to focus on the potential central mechanism of real and non-penetrating sham acupuncture treatments for FD. We will investigate the associations between the clinical efficacy of acupuncture and the brain’s response to determine any differences with those of the sham acupuncture procedure. Additionally, our study is the first trial to focus on the methods of inducing abdominal discomfort experimentally by MA and a meal. As visceral hypersensitivity is one of the major mechanisms for PDS [54], we will investigate the central mechanism of hypersensitivity using fMRI and the mechanism of how acupuncture reduces MA-induced abdominal discomfort in both short-term and longitudinal periods.

FD is classified into two subtypes: PDS and epigastric pain syndrome (EPS). Patients with PDS complain of bothersome fullness and abdominal discomfort after meals, while EPS focuses on an epigastric burning sensation that frequently occurs during fasting [17]. PDS is considered a more appropriate target subject than EPS in this trial because meal ingestion can aggravate PDS symptoms [55], and PDS patients can be more vulnerable to epigastric pressure due to abdominal hypersensitivity [56]. Another reason to choose PDS as a subject in this trial is the large proportion of PDS compared with other subtypes in Korea. Though more EPS patients are detected than PDS patients in the USA due to the tendency to overestimate gastroesophageal reflux diseases [57], more than 70 % of FD patients were designated to the PDS subtypes in Korea [3]. Our previous study [16] also found a more than 60 % PDS subtype ratio; therefore, PDS is considered to represent most FD patients in Korea.

The effect of acupuncture on the hypothalamus and its influence on the autonomic nervous system [58, 59] implies a homeostatic feature, which is thought to be closely related to a TKM perspective and focuses on systemic and holistic treatment. Therefore, metabolism studies are gaining attention in the field of acupuncture trials. One previous study showed the acupuncture effect of reversing the levels of metabolites affected in FD patients, such as leucine/isoleucine and lactate, so that they more closely resemble those of healthy controls [15]. However, this method had several defects, such as a small sample size (six subjects per group), the absence of a sham control, and only profiling polar metabolites by NMR. In the present study, we planned 35 subjects per group as a comparatively large sample, and the change and difference of metabolomic profiles will be investigated by H NMR and MS compared with the sham group.

Several studies have suggested various coping strategies for FD. Patients with FD have been reported to use non-discriminative coping strategies [60], and other studies have identified a problem-focused and emotion-focused coping style for FD patients [61, 62]. The importance of investigating patients’ coping style with FD symptoms is emphasized by the following conditions: 1) a negative appraisal of FD symptoms or anxiety can be a key indicator that differentiates the FD and the normal group [63], 2) the anxiety of FD patients is closely related to the brain’s response to pain [53], and psychological coping can be a good clinical treatment method for FD [64, 65]. Our trial will investigate the influence of coping mechanisms for acupuncture on its treatment effect for FD.

A potential limitation of our study is its small sample size, and this problem will be the focus of future study. One of the main features of our study is that we will investigate central and molecular biological mechanisms simultaneously; therefore, we expect our findings to provide a glimpse of the true mechanism behind acupuncture for FD.

### Trial status

Participant recruitment is currently ongoing.

## Abbreviations

ACC: anterior cingulate cortex
AE: adverse events
AFNI: Analysis of Functional NeuroImages
ANCOVA: analysis of covariance
BOLD: blood oxygenation level-dependent
CES-D: Center for Epidemiological Studies Depression Scale
CONT: continuous pressure stimuli run
CSQ: Coping Strategies Questionnaire
EPS: epigastric pain syndrome
FD: functional dyspepsia
FD-QoL: Functional Dyspepsia-Related Quality of Life
fMRI: functional magnetic resonance imaging
FSL: FMRIB Software Library
GLM: generalized linear model
IRB: Institutional Review Board
MA: modified pressure algometer
MNI: Montreal Neurological Institute
NDI: Nepean Dyspepsia Index
NDI-K: Nepean Dyspepsia Index - Korean version
NMR: nuclear magnetic resonance
NRS: Numeric Rating Scale
OPLS-DA: orthogonal projections to latent structure-discriminant analysis
PBS: perception of bodily sensation
PCA: principal component analysis
PDS: postprandial distress syndrome
PET: positron emission tomography
PLS-DA: partial least squares-discriminant analysis
PR: proportion of responders
REST: resting state run
STAI: State-Trait Anxiety Inventory
STRICTA: STandards for Reporting Interventions in Clinical Trials of Acupuncture
TASK1: pressure stimuli run before acupuncture
TASK2: pressure stimuli run after acupuncture
TE: echo time
TKM: traditional Korean medicine
TR: repetition time
UPLC-Q/TOF MS: ultra-performance liquid chromatography quadrupole/time of flight mass spectrometry
VAS: visual analog scale
VIP: variable influence on projection
WCQ: Ways of Coping Questionnaire
